# Optimizing random skin biopsies: a review of techniques and indications for intravascular large B-cell lymphoma

**DOI:** 10.1007/s12185-024-03757-5

**Published:** 2024-04-02

**Authors:** Naoko Enzan, Akihiro Kitadate, Michihiro Kono

**Affiliations:** 1 Department of Dermatology and Plastic Surgery, Akita University Graduate School of Medicine, 1-1-1 Hondo, Akita, 010-8543 Japan; 2 Department of Hematology, Nephrology and Rheumatology, Akita University Graduate School of Medicine, Akita, Japan

**Keywords:** Incisional biopsy, Intravascular large B-cell lymphoma, Malignant lymphoma, Punch biopsy, Random skin biopsy

## Abstract

Intravascular large B-cell lymphoma (IVLBCL), a rare subtype of malignant lymphoma, is diagnosed by observation of intravascular proliferation of tumor cells in samples taken from affected organs. However, diagnosis of IVLBCL is usually difficult due to the lack of mass formation. IVLBCL may be fatal when the diagnosis is delayed, so an accurate early diagnosis is the key to successful treatment. Random skin biopsy (RSB), in which specimens are sampled from normal-appearing skin, has been reported as useful. However, the specific method of RSB remains controversial, with individual institutions using either the punch method or the incisional method. Research has shown that the incisional method has higher sensitivity than the punch method. We discuss whether this difference might owe to the collection of punch specimens from an insufficient depth and whether the punch method might result in false negatives. For RSB, we recommend taking specimens not only from normal-appearing skin, but also from any lesional skin, because lesions may reflect micro IVLBCL lesions. To ensure accurate diagnosis, both dermatologists and hematologists should know the proper method of RSB. This review summarizes the appropriate biopsy method and sites for RSB.

## Introduction

Intravascular large B-cell lymphoma (IVLBCL) is a subtype of malignant lymphoma that is characterized by the proliferation of tumor cells within vessels [1]. Because of its unspecific symptoms, IVLBCL is always challenging to diagnose. Therefore, many cases have been diagnosed by autopsy [2–5]. As the tumor cells can invade any organ, IVLBCL had been diagnosed by taking samples from affected organs, such as the kidneys, lungs, and brain [2, 6–10]. However, these biopsies are usually difficult because of the deteriorated condition of the patient and the rapid progression of the disease. In addition, due to the lack of lymphadenopathy and mass formation, it is difficult to determine an adequate biopsy site. A skin biopsy is easier and less invasive. Sampling from normal-appearing skin, called random skin biopsy (RSB), has been reported useful for diagnosing IVLBCL [2, 11–14]. Patients with IVLBCL are mostly diagnosed from bone marrow and/or skin biopsy [15]. The share of diagnoses from bone marrow (20.5%) is less than that from skin (74.4%) [16]. Furthermore, the number of patients diagnosed with IVLBCL by skin biopsy has been increasing recently. Only 7.4% of patients were diagnosed with IVLBCL from the skin in 2007 [17]; however, more than half of patients with IVLBCL were diagnosed from the skin around 2020 [14, 18]. Hence, the skin is an important diagnostic site for IVLBCL. Although IVLBCL has been a fatal disease, at 2008 study found that patients treated with rituximab showed improved outcomes [19, 20]. Moreover, the progression-free survival and overall survival at 2 years were reported to be 76% and 92% in 2020, respectively, owing to the use of rituximab [18]. Therefore, diagnosing IVLBCL early promises to increase the likelihood of successful treatment. In this review, we provide recommendations on the optimal method of RSB. Matsue et al., who conducted a large case series of RSB, used “RSB” to refer to sampling not only from normal-appearing skin but also from visible skin lesions. Our review follows this terminology [2, 14, 16].

## IVLBCL subtypes

There are three subtypes of IVLBCL: a classical subtype, a hemophagocytic subtype, and a cutaneous subtype [1, 12]. Patients with the hemophagocytic subtype show a typical clinical hemophagocytic syndrome. The cutaneous subtype presents as single or multiple skin lesions with negative systemic staging [1, 21]. Ferreri et al. reviewed 38 patients with IVLBCL and found that cutaneous lesions were the dominant presenting features in 15 patients. Ten of those 15 patients had recognizable lesions restricted to the skin, a condition that was named the cutaneous variant of IVLBCL [21]. Most cases of IVLBCL show lesion that are not limited to the skin and with skin lesions usually being absent [12, 21]. The incidence rate of the cutaneous subtype is much lower in Asia than in the West (3% vs. 24%) [22]. The cutaneous subtype is mainly observed in younger women. Furthermore, almost all patients with the cutaneous subtype showed an excellent performance status and rarely had B symptoms, a set of symptoms including fever above 38 °C, drenching night sweats, and weight loss of more than 10% of body mass. Ferreri et al. hypothesized that the cutaneous subtype shows a better prognosis due to the easier diagnosis of the cutaneous lesions or to biological differences from other subtypes [21]. IVLBCL usually lacks both visible skin lesions and specific symptoms, so except for the cutaneous subtype, IVLBCL is prone to late diagnosis or misdiagnosis, which results in worse prognosis than for the cutaneous subtype [3, 5]. Therefore, the early and accurate diagnosis of IVLBCL other than the cutaneous subtype is especially important.

## History of RSB

IVLBCL lesions have been observed in almost every organ, including the skin, in autopsy cases [2, 23]. Before the establishment of RSB, no study had reported IVLBCL diagnosed from normal-appearing skin, although there had been IVLBCL cases that were diagnosed from skin rashes [24–27]. Demirer et al. were the first to report a case of IVLBCL diagnosed by lip biopsy without skin lesions, in 1994 [23]. A case of IVLBCL diagnosed by RSB was reported in 2003 [28]. Subsequently, the usefulness of RSB was reported [11, 13, 29–31]. Asada et al. reported six cases of IVLBCL diagnosed by RSB [11]. They examined 26 specimens obtained from six patients with IVLBCL and found that 23 of the specimens (88.5%) included IVLBCL lesions. As the skin biopsy was easier than biopsy from other organs, they concluded that if IVLBCL is suspected, RSB should be considered. Although bone marrow aspiration and biopsy were performed in all cases in that study, no tumor cells were found within vessels in those specimens [11]. Bone marrow biopsy continues to be widely performed to screen for IVLBCL; however, its sensitivity has been low [16, 32]. The marrow pattern of IVLBCL is categorized in three patterns: an intrasinusoidal pattern with or without minimal extravasation, an intrasinusoidal pattern with substantial scattered/interstitial extravasation, and a nodular/diffuse pattern. Of these patterns, the intrasinusoidal marrow infiltration pattern with or without minimal extravasation is diagnostic for IVLBCL [32]. Matsue et al. reported that 18 patients (60%) showed bone marrow infiltration among 30 patients with IVLBCL diagnosed by RSB. Nevertheless, only five patients showed an intrasinusoidal marrow infiltration pattern in the bone marrow. Hence, the sensitivity of bone marrow biopsy was only 16.7% [32]. Accordingly, even if IVLBCL is diagnosed by RSB, the bone marrow infiltration may be incongruous with the skin pathology. Thus, some patients suspected of having IVLBCL who receive only bone marrow biopsy without RSB may be misdiagnosed as negative for IVLBCL. In clinical practice, patients suspected of having IVLBCL are evaluated by RSB and bone marrow biopsy in the initial workup. In cases where RSB is positive with negative bone marrow biopsy, the diagnosis of IVLBCL is made [11, 16]. Owing to its convenience, RSB has gradually become widespread. However, no unified RSB method has been established, and the procedure varies among institutions. Furthermore, indicators of which patients can most benefit from RSB have not been established.

## The appropriate RSB method

**Fig. 1:**
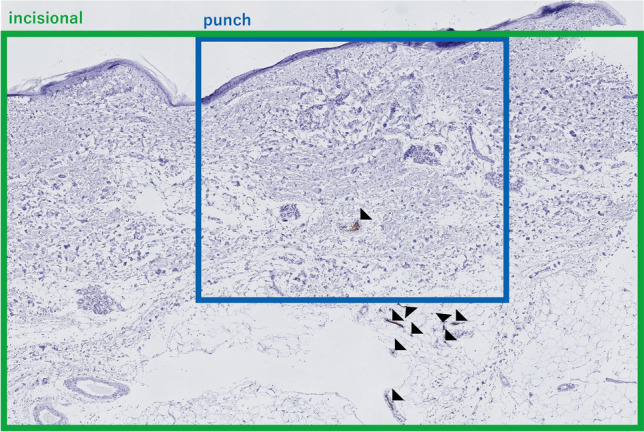
A specimen of intravascular large B-cell lymphoma sampled by incisional random skin biopsy. Tumor cells stained by CD20 immunostaining are predominantly distributed within the vessels in the subcutaneous fat tissue. Black arrows indicate the affected vessels. The blue-framed area shows the depth and width of the specimen that can be sampled by the punch method. The specimen that can be taken by the incisional method is indicated by the green-framed area (× 40; CD20 immunostaining)

RSB has been performed by two methods: a punch method and an incisional method. The punch method is easier and less invasive than the incisional method. However, the incisional method can sample specimens deeper and wider than the punch method. No unified method for RSB has been established [11, 13, 29, 30, 33, 34]. Asada et al. reported that the affected vessels in specimens are distributed predominantly in the subcutaneous fat tissue [11]. We previously examined the depth of the affected vessels in 82 specimens from 25 patients with IVLBCL diagnosed by incisional RSB [35]. IVLBCL lesions were significantly more numerous in subcutaneous fat tissue than in the dermis. Furthermore, among the 25 patients with IVLBCL, 19 (76%) showed dermal and subcutaneous invasion, and the remaining 6 (24%) showed only subcutaneous invasion. All 77 specimens with IVLBCL lesions among the 82 investigated specimens exhibited subcutaneous invasion. We also found that 14 of 38 (37%) specimens in which the affected vessels presented only in subcutaneous fat tissue showed a minimum depth exceeding 5 mm from the skin surface to the lesion [35]. Moreover, we conducted a study that compared specimen depth for punch RSB versus incisional RSB. The median depth of the punch specimens was found to be less than that of the incision specimens. In addition, approximately 40% of the specimens obtained by the punch method measured less than 5 mm [36]. Hence, a punch biopsy may result in false negatives. Two cases of IVLBCL diagnosed by incisional RSB after the failure of punch RSB due to an insufficient amount of subcutaneous fat tissue were reported [34, 37]. From the above, it is clear that the affected vessels are predominantly distributed in the subcutaneous fat tissue [11, 35]. Thus, the punch method could be insufficient for detecting tumor cells in IVLBCL (Fig. 1). Supporting this idea, the sensitivity of punch RSB was found to be low, at 0–50% [38, 39], whereas Matsue et al. reported the sensitivity of incisional RSB to be high (77.8%) [14]. Since the punch method is the predominant method in Western countries, the sensitivity of RSB in Western countries might be lower [34, 38–41]. Maekawa et al. reported a case series of RSB that used either an incisional or a punch method. All nine patients with RSB-positive IVLBCL underwent incisional RSB in the Maekawa report [33]. In summary, the sensitivity of punch RSB was found to be lower than incisional RSB (Table 1). Consequently, sufficient sampling depth is important for detecting IVLBCL lesions.

**Table 1:** Summary of a case series on RSB for suspected IVLBCL

Author (year)	Method	Sensitivity (%)	Specificity (%)
Maekawa et al. [33] (2015)	Both	81.8	100
Cho et al. [38] (2017)	Punch	0	100
Rozenbaum et al. [39] (2021)	Punch	50	100
Matsue et al. [14] (2019)	Incisional	77.8	98.7

*IVLBCL* intravascular large B-cell lymphoma, *RSB* random skin biopsy

To determine how deep the biopsy specimen should be, Maekawa et al. examined the pathology of patients with incisional RSB-positive IVLBCL. Of samples from nine patients with incisional RSB-positive IVLBCL, samples from 8 patients included a whole layer of subcutaneous fat tissue with the deep fascia. The Maekawa group found that there were no specimens in which the affected vessels were limited to the lower layer of subcutaneous fat tissue [33]. Although it seems more diagnostically effective to sample specimens that include the deep fascia, which could contain many more vessels, it is difficult to perform such a sampling in patients who have thrombocytopenia and a deteriorating condition. Furthermore, it is difficult to ensure hemostasis in the fascia, which has a rich blood flow. Moreover, the Maekawa group reported that there were significantly more atypical cells in the upper layer than in the lower layer of subcutaneous fat tissue [33]. Accordingly, sampling specimens that include the superficial fascia can be regarded as reasonable. For deeper sampling, Winge et al. proposed a telescoping method in which a small punch biopsy is telescoped into a larger punch biopsy defect [42]. They concluded that this method could obtain adequate subcutaneous fat tissue. With regard to hemostasis, however, a narrow operative field is associated with a higher risk of bleeding, especially in IVLBCL patients with thrombocytopenia. Therefore, further studies are warranted. An incisional biopsy can sample specimens not only to a much greater depth, but also to a greater width than the punch method. Although the difference between these widths may also contribute to the high sensitivity of incisional RSB, no study has yet addressed this question.

The greater is the number of specimens, the higher is the sensitivity of the RSB. However, complications from bleeding are more likely in IVBCL patients with thrombocytopenia. Moreover, obtaining numerous specimens is often painful for the patient. We previously reported high sensitivity and specificity for incisional RSB that obtained specimens from at least three separate sites [14]. Moreover, it is desirable to take an adequate volume of specimens from fat-rich areas. Consequently, it is reasonable to obtain specimens from at least three separate fat-rich areas of the skin, such as the thigh or abdomen for the maximum sensitivity and the minimum risk of complications.

## Appropriate sites for RSB: normal-appearing skin vs. visible skin lesions

**Fig. 2:**
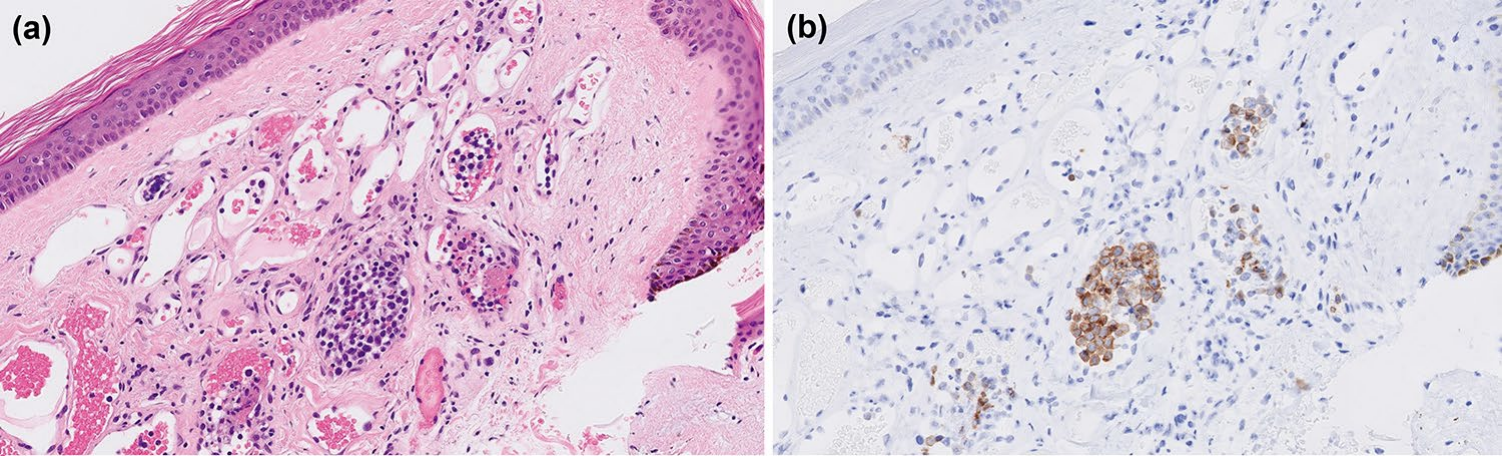
Histopathological and immunohistochemical findings of intravascular large B-cell lymphoma sampled from a cherry angioma. (**a**) The capillaries are filled with atypical lymphoma cells (× 100; hematoxylin and eosin). (**b**) CD20-positive tumor cells have proliferated within the vessels (× 100; CD20 immunostaining)

Although only 14.3% of patients with IVLBCL were found to have skin lesions, visible skin lesions associated with IVLBCL are known to present diversely as nodules, plaques, erythema, and telangiectasia [16, 43]. There is the question of whether to look for visible skin lesions as targets for sampling before RSB. Arai et al. compared the positivity rates for skin lesions to those for normal-appearing skin in IVLBCL. They concluded that neoplastic cells may be present more frequently in skin lesions than in normal-appearing skin [44]. Several reports have recommended biopsies from visible skin lesions, especially for cherry angiomas [45–48]. Ishida et al. reported an IVLBCL patient who had no affected vessels in the specimens taken from normal-appearing skin and who was finally diagnosed from a cherry angioma [46]. We also encountered an IVLBCL case with tumor cells within the vessels in a cherry angioma (Fig. 2). In general, cherry angiomas arise in everyone, and their numbers increase with age. Because of their abundant capillaries, tumor cells become trapped in those vessels. Thus, a cherry angioma may show higher rates of positivity than those of normal-appearing skin in RSB [47]. On the other hand, Saurel et al. described the case of a cutaneous variant of IVLBCL with nodules and spider angiomas that mostly disappeared after the completion of therapy. They evaluated the expression of vascular endothelial growth factor and secreted phosphoprotein 1, which are angiogenic factors in tumor cells. As the tumor cells expressed these angiogenetic factors, they hypothesized that these factors might play a role in the formation of pseudohemangiomoformative lesions [49]. Similarly, Weingarten et al. reported a case of IVLBCL with cherry angiomas that disappeared and subsequently reappeared [50]. Thus, the unusual progress of cherry angiomas in patients with IVLBCL might be due to angiogenic factors from the tumor cells. However, due to the small number of cases, the cause of cherry angiomas in IVLBCL needs to be further investigated.

As mentioned above, the eruptions in IVLBCL patients can be varied, so thrombophlebitis, vasculitis, and livedo racemose might be included as differential diagnoses for IVLBCL. When those symptoms are seen in IVLBCL patients, they are initially caused by the occlusion of vessels by tumor cells, which consequently activates the coagulation cascade, and thrombosis develops within the lumina of the vessels [43]. If superficial vessels are involved, the clinical pattern may be that of livedo racemose; if vessels in the deep dermis or subcutaneous fat tissue are involved, the clinical lesions mimic those of erythema nodosum or nodular vasculitis [43]. Essentially, skin lesions in IVLBCL may reflect IVLBCL lesions in the micro-findings. Hence, we recommend taking specimens not only from normal-appearing skin, but also from any clinically recognized skin lesions. Several patients with IVLBCL have happened to be diagnosed from skin lesions, such as peau d’orange [26, 27], indurated dermal plaques with overlying telangiectasia [25, 27], subcutaneous nodules [24, 27], and red macules with nodules [24]. In addition, a case of IVLBCL diagnosed from skin lesions detected by dermoscopy was recently reported [51]. Dermoscopy enabled the identification of appropriate biopsy sites by showing telangiectasia that was too faint to be recognized by the naked eye. Hence, to identify the appropriate biopsy sites, normal-appearing skin should be reconfirmed using dermoscopy to find subtle telangiectasia. In the absence of skin lesions, positron emission tomography (PET)/computed tomography (CT) may help determine biopsy sites before RSB. In fact, Matsukura et al. reported a case of IVLBCL that was eventually diagnosed by the re-biopsy of an abnormal uptake site of PET/CT after an initially negative RSB [52].

## The selection of patients for whom RSB is appropriate

The prevalence of patients evaluated by RSB who are actually diagnosed with IVLBCL varies widely from study to study. Matsue et al. examined patients who underwent RSB for suspected IVLBCL. Among the 111 patients who underwent RSB, 33 patients were finally diagnosed with IVLBCL [14]. In contrast, Rozenbaum et al. reported that 12% of patients receiving RSB were eventually found to have the disease [39]. Similarly, we reported that patients with IVLBCL comprised 11% of patients who had undergone RSB previously [36]. From these facts, appropriate selection criteria for RSB should be provided, while any oversight is avoided. Matsue et al. proposed six predictors for positive RSB: (1) unexplained fever (≥ 38 °C), (2) altered consciousness, (3) hypoxemia (≤ 95%), (4) thrombocytopenia (< 120 × 10^3^/μL), (5) high serum lactate dehydrogenase (LDH)(> 800 U/L), and (6) high soluble interleukin-2 receptor (sIL-2R)(> 5000 U/mL). The more of these predictors that were met, the higher was the rate of positive RSB [14]. Among these items, Sumi–Mizuno et al. focused on LDH and sIL-2R. If both parameters are normal, the Sumi–Mizuno group consider that RSB should not be performed, to avoid unnecessary biopsies [53]. These predictors would be useful in selecting patients for whom RSB is appropriate.

## Complications of RSB

Despite RSB being a less-invasive method, patients can still have complications, such as bleeding. It was reported that a patient with IVLBCL experienced hemorrhagic shock after an incisional RSB, and it was proposed that a punch biopsy would be preferable as an initial step to prevent accidental bleeding in IVLBCL patients with thrombocytopenia. It was also proposed that if the initial RSB is negative, a second RSB may be considered at an alternative site [33]. However, repeated biopsies are difficult because of the patient’s deteriorating condition and rapid disease progression. To minimize hemorrhagic risk, it is important to correct the coagulopathy and transfuse platelets before RSB [35]. No severe complications other than bleeding have been reported in RSB. With adequate preparation before RSB, this complication can be avoided.

## Conclusion

RSB plays an important role in diagnosing IVLBCL. Even patients in deteriorated condition are able to receive RSB because of its lower invasiveness. Moreover, unlike biopsies from other organs, RSB can be performed at the bedside. Even so, we must recognize the risk of this method. Furthermore, because an inappropriate RSB can result in a false negative, it is important to perform incisional RSB appropriately. For an accurate diagnosis of IVLBCL, we should plan to obtain at least three specimens from fat-rich areas. Also, we should examine the whole body to find skin manifestations, such as cherry angioma and detect faint lesions including telangiectasia by dermoscopy, and we should consider PET/CT before RSB execution. If there are any skin lesions that might contain IVLBCL lesions, we should consider RSB not only from normal-appearing skin, but also from the lesional skin. RSB has become a popular method; however, unnecessary RSBs might still be performed. Thus, we recommend using the predictive criteria of positive RSB mentioned above. IVLBCL can be cured if it is diagnosed early and accurately. We strongly expect that an appropriate method of performing RSB will gain acceptance as a common diagnostic technique worldwide.

## Data Availability

Data sharing is not applicable as no datasets were generated or analyzed.
